# Improving the efficacy of immunotherapy for colorectal cancer: Targeting tumor microenvironment-associated immunosuppressive cells

**DOI:** 10.1016/j.heliyon.2024.e36446

**Published:** 2024-08-16

**Authors:** Daoyang Zou, Xi Xin, Yunxian Xu, Huangzhen Xu, Linyan Huang, Tianwen Xu

**Affiliations:** a The Second Affiliated Hospital of Fujian Medical University, Quanzhou, 362000, China; b Ganzhou People's Hospital, Ganzhou, 341000, China

## Abstract

Currently, immune checkpoint inhibitors (ICIs) have changed the treatment paradigm for many malignant tumors. As the most common digestive tract malignancy, colorectal cancer (CRC) shows a good response to ICIs only in a small subset of patients with MSI-H/dMMR CRC. In contrast, patients with MSS/pMMR CRC show minimal response to ICIs. The results of the REGONIVO study suggest that targeting the tumor microenvironment (TME) to improve immunotherapy outcomes in MSS/pMMR CRC patients is a feasible strategy. Therefore, this article focuses on exploring the feasibility of targeting the TME to enhance immunotherapy outcomes in CRC, collecting recent basic research on targeting the TME to enhance immunotherapy outcomes in CRC and analyzing ongoing clinical trials to provide a theoretical basis and future research directions for improving immunotherapy outcomes in MSS/pMMR CRC.

## 1 Introduction

Colorectal cancer (CRC) is the third most common cancer and the second leading cause of cancer-related deaths globally [1]. It is also the most common digestive tract malignancy. Recent data from the United States shows that CRC remains the third most common cancer and the second leading cause of cancer-related deaths [2]. Although the overall incidence of CRC has been declining in recent years, there has been a rapid increase in cases among younger individuals and in advanced stages [2]. Similar to most cancers, the main treatment modalities for CRC include chemotherapy, radiation therapy, and surgery. The choice of treatment depends on the location and stage of the tumor. Surgery is the preferred treatment for localized CRC (stage I-III) [3], and adjuvant chemotherapy is recommended for patients with lymph node-positive (stage III) disease to eradicate potential residual cancer cells and reduce the risk of recurrence [4]. For advanced or metastatic CRC, a multidisciplinary approach is generally employed. In 2013, cancer immunotherapy was recognized as the most significant scientific breakthrough of the year by the journal Science [5]. Over the past decade, immune checkpoint inhibitors (ICIs) have completely changed the treatment paradigm for many malignant tumors. However, ICIs have shown promising responses in only a small subset of patients [6], with less than 10 % of MSI-H/dMMR CRC patients achieving clinical efficacy rates of only 43%–65 % [7,8]. Most patients with MSS/pMMR CRC have minimal response to immunotherapy [9,10]. Therefore, exploring strategies to improve immunotherapy outcomes in CRC has become a hot research topic.

Previous studies have shown that solid tumors can exhibit two phenotypes in response to immunotherapy. One phenotype is the “hot tumor” (immune-inflamed) which responds to immunotherapy and is characterized by infiltrating T lymphocytes. The other phenotype is the “cold tumor” (immune desert/immune excluded) which does not respond to immunotherapy and is characterized by a lack of or exclusion of T lymphocyte infiltration within the tumor tissue [11]. For MSS/pMMR CRC, it is considered a “cold tumor” in traditional terms due to its low tumor mutation burden and lack of immune cell infiltration [10,12]. However, emerging evidence suggests that the infiltration of T lymphocytes alone cannot completely predict the response to immunotherapy. Poorly infiltrated tumors can still exhibit good responses to ICIs, while highly infiltrated tumors may have poor responses [6,13,14]. Many studies now believe that different tumor microenvironments (TME) contribute to different outcomes of ICIs treatment [15]. TME refers to the cellular environment in which the tumor or tumor stem cells exist. It includes various immune infiltrating cells (such as macrophages, dendritic cells, and lymphocytes) around the tumor, blood vessels, extracellular matrix (ECM), fibroblasts, bone marrow-derived suppressor cells, cytokines, and signaling molecules [[16], [17], [18], [19]]. Increasing evidence suggests that TME plays a critical role in tumor progression, immune escape, and response to immunotherapy [[20], [21], [22]]. Therefore, modulating the TME to improve the efficacy of ICIs is a promising approach [[23], [24], [25], [26]]. This study aims to explore potential ways to improve immunotherapy outcomes in CRC by targeting the TME through reviewing recent research on targeting TME-related immune inhibitory cells in CRC. It provides directions for future research in improving immunotherapy outcomes in CRC.

### 1.1 Tumor-associated macrophages (TAMs)

Tumor-associated macrophages (TAMs) are considered to be one of the most closely associated tumor-infiltrating immune cells in the tumor cell and TME interaction [27,28]. TAMs are believed to be a key driving factor in creating an immunosuppressive microenvironment by recruiting through colony-stimulating factor (CSF), transforming growth factor-beta (TGF-β), chemokines (such as CCL2, CCL4), vascular endothelial growth factor (VEGF), and angiopoietin-5 [29,30]. Macrophages have a high degree of plasticity and can be activated into M1 (anti-tumor) or M2 (tumor-promoting) phenotypes depending on different microenvironmental stimuli [[31], [32], [33]]. More and more studies have shown that TAMs promote tumor growth, stimulate angiogenesis, enhance tumor cell migration and invasion, and suppress anti-tumor immunity [[34], [35], [36]]. Therefore, modulating TAMs and reshaping the immune microenvironment to improve immunotherapy outcomes in CRC is a feasible strategy.

Studies have shown that the loss of miR-146b promotes the development of TAMs towards the M2 phenotype, inhibits T-cell infiltration, and facilitates the transformation of an immune-suppressive TME. Upregulating miR-146b enhances the anti-tumor activity of anti-PD-1 immunotherapy [37]. Silencing BST2 significantly inhibits TAM polarization towards the M2 phenotype and restricts tumor proliferation in a CRC mouse model [38]. Applying self-assembling traditional Chinese medicine nanodrugs can regulate TAMs to repolarize from the tumor-promoting M2 phenotype to the anti-tumor M1 phenotype, the proportion of M1 phenotype significantly increased from 8.8 % to 31.0 %, while the proportion of M2 phenotype decreased 68.7 %–43.0 %, effectively reshaping the immune-suppressive TME and mobilizing innate and adaptive immunity to inhibit tumor progression [39]. The use of bidirectional anisotropic Pd nanoclusters (Pd-HA + Pd-M@R NPs) specifically taken up by TAMs induces their conversion into the M1 phenotype. In the control group, the M1/M2 ratio was only 0.15. However, under the effect Pd-HA + P-M@R NPs, this ratio reached 1.1, an increase of nearly 7-fold, thereby reversing tumor immunosuppression [40]. Studies have also shown that inhibiting tumor-derived immunoglobulin-like transcript 5 (ILT5) can reduce M2 polarization of TAMs, restore immune-responsive T cells, restrict CRC progression, and improve the response to immunotherapy [41]. Combining multikinase inhibitor foretinib with anti-PD-1 antibody treatment significantly inhibits tumor growth in a CRC mouse model, Foretinib (67.97 % inhibition) and PD-1 (76.69 % inhibition) individually inhibited tumor growth, but the inhibitory effect was significantly enhanced after the combination treatment (For + PD-1, 98.05 %). reduces the percentage of TAMs, and inhibits their polarization towards the M2 phenotype, thereby reshaping the TME and enhancing anti-tumor immunity [42]. LncRNA PTTG3P promotes the polarization of TAM to M2 phenotype [43]. Therefore, modulating TAMs to convert them from the M2 to M1 phenotype or inhibiting TAM polarization towards the M2 phenotype is a feasible approach to improve immunotherapy outcomes in CRC.

Specifically targeting certain pathways to unleash the anti-tumor activity of TAMs is also a viable strategy. The specific knockout of AP-2α significantly enhances the phagocytic activity of TAMs and inhibits colorectal cancer progression [44]. Blocking the CD47-SIRPα interaction using ZL-1201 enhances the tumor phagocytic ability of TAMs, reshapes the TME, and improves the response to immunotherapy [45]. Research has also shown that M2 macrophage-derived exosomal miR-155-5p promotes immune escape in colorectal by regulating ZC3H12B. Therefore, inhibiting the production of immunosuppressive factors by M2 macrophages is an approach to improve the efficacy of immunotherapy for CRC [46].

Targeting colony-stimulating factor 1 receptor (CSF1R) significantly reduces M2 TAM infiltration and increases CD8 T cell infiltration in CRC tumor tissue, effectively inhibiting tumor growth and metastasis, and improving the response to immunotherapy [[47], [48], [49]]. Inactivating chemokines CCL2 and CCL7 limits TAM recruitment [50]. Down-regulation of LncRNA H19 expression can inhibit M2 macrophage infiltration [51]. Therefore, selectively blocking cytokines involved in TAM recruitment to inhibit TAM tumor infiltration and improve the tumor immune microenvironment is a promising approach to enhance immunotherapy outcomes in CRC.

**Fig. 1:**
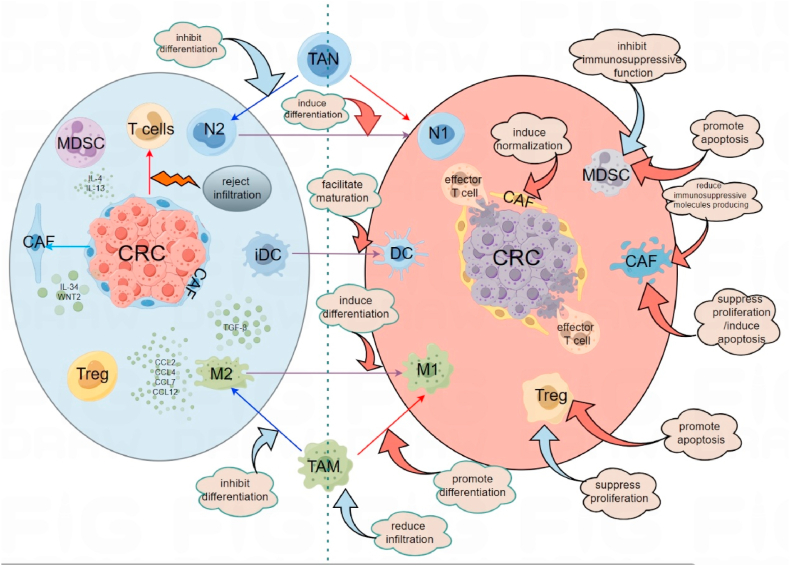
Approaches to improving immunotherapy efficacy in CRC through targeted modulation of TME-related immunosuppressive cells.

In summary, modulating TAMs to convert them from the M2 to M1 phenotype, inhibiting TAM polarization towards the M2 phenotype, inducing TAM repolarization towards the M1 phenotype to reactivate anti-tumor functions, and blocking TAM recruitment-related chemokines to reduce M2 TAM tumor infiltration are feasible ways to target TAMs and improve immunotherapy outcomes in CRC (Fig. 1). Promising results have been achieved in preclinical studies. Therefore, targeting TAMs to improve immunotherapy outcomes in CRC is a hopeful strategy.

### 1.2 Cancer-associated fibroblasts (CAFs)

Cancer-associated fibroblasts (CAFs) are one of the most important stromal cell components in the TME, and they exhibit biological heterogeneity in terms of cell origin, phenotype, and function [52,53]. The majority of studies have shown that CAFs play a role in promoting tumor progression. CAFs remodel the extracellular matrix (ECM), weaken the tumor immune microenvironment, and reprogram cancer cell metabolism, thereby promoting tumor metastasis and immune evasion [[54], [55], [56], [57]]. Studies have shown a close relationship between CAFs and CRC prognosis, with genes associated with CRC recurrence and poor prognosis being upregulated primarily in CAFs rather than tumor cells [56]. Therefore, targeting CAFs to improve immunotherapy outcomes in CRC is a hopeful approach.

Research has shown that CAFs promote PD-L1 expression in CRC tumor cells through Akt phosphorylation [58]. Thus, targeting anti-CAF therapy may improve immunotherapy outcomes in CRC. The immune resistance caused by CAFs inhibiting the infiltration of CD8 T cells can be overcome by targeting CAFs and inhibiting the NOX4 enzyme, which leads to the “normalization” of CAFs. In mouse models, the survival period of the combination group was significantly extended (58 days vs 22 days) [59]. The chemokine CXCL12, mainly secreted by CAFs in many solid tumors [60], is involved in immune suppression by recruiting specific immune cell populations [61]. A ketogenic diet reduces the production of CXCL12, improves the immune suppressive microenvironment in CRC tumor-bearing mice, and enhances the efficacy of immunotherapy [62]. WNT2 secreted by CAFs inhibits the anti-tumor T-cell response mediated by dendritic cells (DCs) through the SOCS3/p-JAK2/p-STAT3 signaling pathway. Anti-WNT2 monoclonal antibodies increase the activity of DCs in transplanted tumors in CRC mice, significantly improving the therapeutic efficacy of PD-1 antibodies(p = 0.0012) [63]. Therefore, inhibiting the production of immune-suppressive molecules associated with CAFs may be a feasible approach to improve immunotherapy outcomes in CRC.

Platelet-derived growth factor receptor (PDGFR) is highly expressed in CAFs in the CRC stroma [64]. In a CRC mouse model, the combination of PDGFR inhibitor Dasatinib and anti-programmed cell death-1 (PD-1) antibody has been shown to suppress CAF proliferation, promote immune cell infiltration, activate anti-tumor immunity, and induce tumor regression [65]. Recombinant IL-34 is highly expressed in CRC tissue and promotes CAF proliferation through paracrine and autocrine mechanisms, thereby promoting CRC progression and metastasis. Inhibiting IL-34 expression can suppress CAF proliferation [66]. Fibroblast activation protein (FAP) is overexpressed in more than 90 % of CAFs. Vaccination with eNVs-FAP targeting FAP + CAFs significantly reduces CAFs in tumor-bearing mice and reprograms the immune-suppressive TME, thereby inhibiting tumor growth(P = 0.0086) [67]. Therefore, targeting specific receptors or molecules to inhibit CAF proliferation or induce CAF apoptosis and thereby improve the tumor immune microenvironment is a feasible strategy.

Overall, reducing the production of immune-suppressive molecules by CAFs, inhibiting CAF proliferation, or inducing CAF “normalization” may be effective approaches to target CAFs and improve immunotherapy outcomes in CRC (Fig. 1).

### 1.3 Myeloid-derived suppressor cells (MDSCs)

Myeloid-derived suppressor cells (MDSCs) represent a highly heterogeneous population of bone marrow cells that accumulate under chronic inflammatory stimuli in cancer [[68], [69], [70]]. MDSCs MDSCs consist of two main subsets: monocytic MDSCs (M-MDSCs) and polymorphonuclear MDSCs (PMN-MDSCs). M-MDSCs resemble monocytes in phenotypic appearance, while PMN-MDSCs resemble neutrophils [68,70,71]. It is widely believed that MDSCs are the major mediators of immune suppression in the TME and numerous studies have shown a correlation between increased MDSC numbers and poor response to immunotherapy [[72], [73], [74], [75]]. In colorectal cancer, the accumulation of MDSCs enhances their immunosuppressive effects and promotes the development of CRC [76]. Therefore, targeting MDSCs to improve the immune suppressive microenvironment in CRC and enhance the efficacy of immunotherapy may be a feasible strategy.

MDSCs appear to be highly sensitive to PARP inhibition, and studies have shown that using the PARP-1 inhibitor olaparib can downregulate the immunosuppressive function of colon cancer MDSCs and enhance the efficacy of anti-PD-1 immunotherapy. In a mouse model of AOM/DSS-induced colorectal cancer, 78 % of mice (7 out of 9) were cured through the combination treatment [77]. Asparagus polysaccharide induces apoptosis of MDSCs isolated from the spleens of colon cancer mice in a toll-like receptor 4 (TLR4)-dependent manner, after treatment with asparagus polysaccharide for 48 h, the percentage of viable cells decreased from 92.7 in the control samples to 79.8 %. thereby weakening the inhibitory effect on anti-tumor immune responses [78]. Recent research has shown that IL-4 and IL-13 promote the function of M-MDSCs and inhibit anti-tumor immunity in intestinal tumors, suggesting IL-4 and IL-13 could be potential targets for improving CRC immunotherapy [79]. Tadalafil nanoparticle vaccine induces immunogenic cell death of MDSCs and enhances the efficacy of immunotherapy, as demonstrated in a colon cancer model [80]. High ALKBH5 expression induces MDSC accumulation, reduces natural killer cells and cytotoxic CD8^+^ T cell infiltration, and is associated with poor prognosis in CRC. Targeting ALKBH5 to inhibit MDSC accumulation may be a promising strategy to improve the efficacy of immunotherapy [81]. Research has shown that death receptor 5 (DR5) is highly expressed in MDSCs and tumor cells. Targeting DR5 with agonistic anti-DR5 antibodies specifically eliminates MDSCs and induces the aggregation of CD8^+^ T lymphocytes in tumor tissue. In mouse models, the absolute number of MDSCs decreases by approximately 75.6 %. Combined treatment with anti-DR5 and anti-PD-L1 antibodies synergistically inhibits tumor growth in colon cancer mice. In the combination treatment group, 40 % of mice experienced complete tumor regression [82]. The biomimetic nanosystem shPvt1-CM-D, which encapsulates lncRNA Pvt1, leads to approximately a 70 % reduction in the expression of Pvt1 and its downstream target c-Myc in MDSCs. This inhibition improves the function of MDSCs and enhances the tumor immune microenvironment [83]. N-acyl sphingosine amidohydrolase 2 (ASAH2) is highly expressed in infiltrating MDSCs in colon cancer and acts as a survival factor for MDSCs. Targeting ASAH2 induces ferroptosis of MDSCs, reduces MDSC accumulation, increases the activation of tumor-infiltrating cytotoxic T lymphocytes (CTLs), and inhibits tumor growth. Therefore, targeting ASAH2 to induce MDSC ferroptosis is a potential strategy to improve CRC immunotherapy [84]. The enrichment of MDSCs in the tumor microenvironment is an important barrier to the efficacy of anti-PD-1 antibodies in colorectal cancer. Quinic acid inhibits the immunosuppressive functions of MDSCs, reshapes the tumor immune microenvironment, and enhances the therapeutic effect of anti-PD-1 antibodies in colon cancer [85].

These studies suggest that in CRC, targeting MDSCs in the TME to improve the efficacy of CRC immunotherapy should focus on downregulating the immunosuppressive functions of MDSCs or inducing MDSC apoptosis to reduce their accumulation in the TME (Fig. 1).

### 1.4 Regulatory T cells (Tregs)

Regulatory T cells (Tregs) are a functional subgroup of T cells with phenotypic characteristics such as Foxp3, CD25, CD4, and other expression molecules. They were first discovered by Sakaguchi et al., in 1995 [86]. Current research indicates that Tregs not only play an important role in regulating immune suppression in tumor immunity [[87], [88], [89]] but also have an undeniable role in tumor immune evasion [[89], [90], [91], [92]]. Tregs induce immune suppressive TME through the production of pro-inflammatory and immune inhibitory cytokines. In CRC, Foxp3+CD25+CD4^+^ Tregs inhibit anti-tumor immune responses in CRC patients and mediate immune evasion in CRC [93,94]. Studies have shown that Treg cell depletion in colon cancer increases infiltration and proliferation of T cells in tumors [95]. Therefore, targeting Tregs to improve the efficacy of CRC immunotherapy may be a promising strategy.

Treatment with a p38 inhibitor can inhibit immune tolerance induced by CD25(−) Tregs, but the effect of improving the immune microenvironment disappears when CD25(+) Tregs are present. However, after CD25(+) Tregs are absent, treatment with a p38 inhibitor in mice with colon cancer can enhance the anti-tumor immune response induced by immunotherapy. The tumor size in the p38 inhibitor group decreased by 73.6 %, with tumor size measured at 246 ± 129 mm [96] (n = 6, p < 0.0002, *t*-test), compared to the control group with tumor size measured at 929 ± 466mm^396^. Colon cancer cells secrete miR-208b, which delivers to receptor T cells and promotes Treg proliferation by targeting programmed cell death 4 (PDCD4) [97]. Inhibiting the secretion of miR-208b by colon cancer cells may inhibit Treg proliferation and improve the efficacy of colon cancer immunotherapy. The accumulation of lactate supports the presence of Treg cells and promotes tumor immune evasion [98]. CircSOX1 regulates the glycolysis of CRC cells, reducing lactate accumulation and consequently reducing the infiltration of Treg cells [99]. Wang Rui's study showed that in a mouse CRC model, Treg infiltration increased after treatment with a PD-1 inhibitor, and proprotein convertase subtilisin/kexin type 9 (PCSK9) expression was enhanced. Blocking PCSK9 eliminated the increase in Treg cells induced by the PD-1 inhibitor and enhanced the anti-tumor effect of the PD-1 inhibitor in CRC [100]. PI3Kδ plays an important role in the maturation of Foxp3+ Treg cells, and inhibiting PI3Kδ can effectively improve the immune suppressive microenvironment in CRC tumor-bearing mice [49]. Rubiginosin B (RGB) selectively inhibits the differentiation of Treg cells by targeting the calcium-regulated phosphatase-NFAT signaling pathway, reduces the proportion of Treg cells in the tumor, enhances the anti-tumor immune response in colon cancer mice, and inhibits tumor growth [101].

In summary, targeting Tregs in the TME to improve the efficacy of CRC immunotherapy should focus on how to inhibit Treg proliferation or eliminate tumor-infiltrating Tregs (Fig. 1).

### 1.5 Tumor-associated neutrophils (TANs)

Tumor-associated neutrophils (TANs), as a type of innate immune cells, have been found to infiltrate many types of tumors and play a role in either suppressing or promoting tumor growth in different tumor microenvironments [[102], [103], [104]]. Some studies have shown that increased neutrophil infiltration in CRC tumor tissue is associated with poor prognosis [105], while others have indicated that high levels of neutrophil infiltration are associated with improved overall survival in CRC patients [106,107]. The reason for this contradictory phenomenon is that TANs can have different differentiation states, similar to tumor-associated macrophages (TAMs). TANs can be classified into “N1-type” (anti-tumor) and “N2-type” (pro-tumor) [108]. It is currently known that myeloid-derived suppressor cells (MDSCs) can enter tumors and differentiate into TANs [109]. However, due to the lack of specific markers, it is still unclear whether N2-type TANs are recruited MDSCs or if they are neutrophils derived from the blood that undergo phenotypic conversion in the TME. Retrospective clinical studies have shown that SMAD4-negative CRC leads to increased secretion of CXCL1 and CXCL8, which can induce polarization of TANs into the N2 phenotype and promote CRC progression [110]. Therefore, targeting SMAD4 to inhibit the differentiation of TANs into the N2 phenotype may be a feasible way to improve CRC immunotherapy. Studies have found that inhibiting the secretion of TGF-β from CRC tumor cells can reduce the production of immunosuppressive molecules by tumor-associated neutrophils (TANs). Compared to the control group, the treatment group had a 2–3 fold decrease in mRNA levels of the immunosuppressive molecule arg-1. This induction led to the reversion of TANs from the N2 phenotype to the N1 phenotype and suppressed tumor growth of colorectal cancer [111]. This suggests that inducing the differentiation of TANs from the N2 phenotype to the N1 phenotype is a feasible strategy to improve the immune microenvironment in CRC.

Overall, inducing the differentiation of TANs from the N2 phenotype to the N1 phenotype or inhibiting their differentiation into the N2 phenotype may be potential ways to improve the immune microenvironment in CRC (Fig. 1). However, there is currently limited research on TANs in CRC, and more basic research is needed to further clarify the feasibility of targeting TANs to improve the efficacy of CRC immunotherapy.

### 1.6 Dendritic cells (DCs)

Dendritic cells (DCs) are professional antigen-presenting cells that are capable of activating naive T cells. They play a crucial role in inducing adaptive anti-tumor immune responses, and tumor-infiltrating conventional DCs (cDCs) are particularly important [[112], [113], [114]]. Type 1 cDCs (cDC1) are the major subset that can cross-present antigens and activate CD8 T cells through the major histocompatibility complex class I (MHC-I) pathway, and activate CD4 T cells through the MHC-II and CD4 signaling pathway to induce adaptive anti-tumor immune responses [112,115]. On the other hand, cDC 2 can activate Th1, Th2, Th17, and CD8 T cells in vitro and drive anti-tumor CD4 T cell immune responses [116,117]. However, the immunosuppressive TME can cause DCs to differentiate into immunosuppressive regulatory DCs, such as those that secrete TGF-β, which inhibits the activation of cytotoxic T cells and promotes tumor progression [114,118,119]. Research has shown that DCs derived from rat colon cancer tissues not only significantly reduce the induction of T cell proliferation but also induce T cell dysfunction [120]. It has also been found that the immunosuppressive matrix protein-polysaccharide in CRC can exert immunosuppressive effects by inhibiting DC differentiation [121]. Therefore, targeting tumor-infiltrating DCs to improve the efficacy of CRC immunotherapy may be a potential approach.

High expression of CD73 is associated with poor prognosis in CRC and leads to reduced infiltration of CD45^+^ and CD8^+^ immune cells. Patients with high expression of CD73 in tumors have a worse disease-free survival (DFS) compared to those with low expression (48.7 % vs 61.%, = 0.019). Targeting CD73 promotes DC maturation and immune cell infiltration, thus improving the efficacy of CRC immunotherapy [122]. Research has shown that incorporating the photosensitizer zinc phthalocyanine (ZnPc) into extracellular ves (EVs) and using ZnPc-EVs for photodynamic therapy can induce the maturation of dendritic cells [123]. Targeting WNT2 secreted by CAFs restores DC differentiation and DC-mediated anti-tumor T cell responses in a CRC mouse model, enhancing the therapeutic effect of PD-1 antibodies [63]. Cell-free supernatant from CRC cells after chemotherapy induces DC phenotypic maturation through Toll-like receptor 4 (TLR4) and enhances anti-tumor immune responses in CRC-bearing mice [124]. It has also been shown that FOXM1 inhibits DC maturation and promotes colorectal cancer progression. Inhibiting the expression of FOXM1 may release the anti-tumor capacity of DCs and improve the immune microenvironment in colon cancer [125].

These studies suggest that targeting specific molecules or receptors to promote the maturation and differentiation of tumor-infiltrating DCs could improve the immune suppressive microenvironment in CRC and enhance the efficacy of immunotherapy (Fig. 1).

### 1.7 Advances in clinical research on improving the immunotherapy effects of CRC by targeting the TME

The above-mentioned basic research indicates that targeting the TME to improve the immunotherapy effects of CRC by regulating immunosuppressive cells is a promising strategy. Currently, several clinical trials are exploring the feasibility of targeting the tumor microenvironment to improve the immunotherapy effects of CRC (Table 1).

**Table 1:** Clinical trials of targeting the tumor microenvironment to improve the efficacy of CRC immunotherapy.

Therapeutic Agent	Therapeutic Strategy	Therapeutic Agent Description	Cancer Type	Clinical trial status	Clinical Trial Reference (Phase)
Imalumab(BAX69)	alone	macrophage migration inhibitory factor	Malignant solid tumour(including mCRC）	Completed	NCT01765790(Ⅰ) [126]
Pexidartinib(anti-CSF1R)	Durvalumab (Anti-PD-L1) combined with Pexidartinib	CSF1R blockade depletes infiltration of M2 macrophages	Pancreatic or CRC	Completed	NCT02777710(Ⅰ)
Imalumab(BAX69)	BAX69 combined with 5-FU/LV or panitumumab	macrophage migration inhibitory factor	mCRC	Terminated	NCT02448810(Ⅱ)
BMS-813160	BMS-813160 Combined with Chemotherapy or Nivolumab	CCR2/CCR5 antagonists	CRC or pancreatic cancer	Completed	NCT03184870(Ⅰ/Ⅱ)
PB101	alone	modulating TME	Malignant solid tumour(including mCRC）	Recruiting	NCT06075849(Ⅰ)
TTX-030(Anti-CD39)	TTX-030 combined with pembrolizumab or budigalimab and/or Chemotherapy	change TME and promote anti-tumor immune response	Advanced Solid Tumors(including mCRC）	Active， not recruiting	NCT04306900(Ⅰ)
Olaptesed (NOX-A12)	Olaptesed alone and combined with pembrolizumab	Targeting the key chemokine CXCL12 in the TME	Pancreatic or CRC	Completed	NCT03168139(Ⅰ/Ⅱ)
DS-8273a	DS-8273a combined with nivolumab	Anti-DR5 antibody	CRC	Terminated	NCT02991196(Ⅰ)
SX-682	SX-682 combined with nivolumab	CXCR1/2 Inhibitor	mCRC (MMS)	Recruiting	NCT04599140(Ⅰ/Ⅱ)
Camidanlumab Tesirine(ADCT-301)	ADCT-301 alone and combined with pembrolizumab	Inhibition of CD25 overexpressing Treg cells	Advanced Solid Tumors (including mCRC)	Terminated	NCT03621982(Ⅰ)
AMG 228	Alone	Monoclonal antibody targeting GITR	Advanced Solid Tumors (including mCRC)	Terminated	NCT02437916(Ⅰ) [127]
Oleclumab(MEDI9447)	Oleclumab alone and combined with durvalumab	Anti-CD73 monoclonal antibody	Advanced Solid Tumors (include mCRC)	Completed	NCT02503774(Ⅰ)

Imalumab (BAX69) is a macrophage migration inhibitory factor. A phaseⅠclinical trial (NCT01765790) evaluated the safety, tolerability, pharmacokinetics, and anti-tumor activity of imalumab in advanced cancer patients. The results showed that out of the 39 evaluated patients, 13 had stable disease, which preliminarily assessed the feasibility of targeting macrophage migration inhibitory factor as a therapeutic target for cancer [126]. However, a phase II clinical trial (NCT02448810) combining imalumab with chemotherapy or panitumumab in CRC patients failed, possibly due to the lack of combination with immunotherapy. Pexidartinib, a CSF1R inhibitor, has been studied in clinical research (NCT02777710) in combination with Durvalumab for the treatment of CRC patients with completed enrollment and await results.

Preclinical studies have shown that Death Receptor 5 (DR5) is highly expressed in MDSCs, and targeting DR5 with an agonistic anti-DR5 antibody can specifically eliminate MDSCs. The combination of anti-DR5 antibody and anti-PD-L1 antibody has synergistic anti-tumor effects in colon cancer mice [82]. DS-8273a, an anti-DR5 antibody, has been studied in a clinical trial (NCT02991196) in combination with nivolumab for the treatment of CRC patients, but the final results have not been reported yet.

Preclinical studies have demonstrated that Tregs play a role as immune suppressors in tumors, with CD25 commonly overexpressed on Tregs. Previous clinical studies targeting CD25 to eliminate Tregs and improve the effectiveness of immunotherapy have shown certain clinical benefits in patients with renal cell carcinoma and breast cancer [128,129]. Camidanlumab Tesirine (ADCT-301) is a CD25 overexpression inhibitor, and a recent clinical trial (NCT03621982) has used ADCT-301 in combination with pembrolizumab for the treatment of advanced solid tumors, including mCRC. The study has completed enrollment and awaits final results. Glucocorticoid-induced tumor necrosis factor receptor (GITR) is expressed in both regulatory T cells (Tregs) and effector T cells. When the antibody binds to GITR on Tregs, the GITR pathway can lead to the depletion of Treg cells, and targeted GITR combined with Nivolumab treatment has been successful in the treatment of advanced solid tumors [130]. AMG 228, which targets GITR, has been studied in a clinical trial (NCT02437916) as a monotherapy for the treatment of advanced solid tumors, including mCRC. The results showed that AMG 228 has good safety, but no significant anti-tumor activity was observed [127], possibly due to the lack of combination with immunotherapy.

Preclinical studies have shown that high expression of CD73 is associated with poor prognosis in CRC, and targeting CD73 to promote DC maturation and immune cell infiltration may improve the effectiveness of CRC immunotherapy [122]. MEDI9447 is a specific human monoclonal antibody targeting CD73, and studies in mouse tumor models have shown broad immune modulatory effects [131]. A clinical trial (NCT02503774) using MEDI9447 in combination with durvalumab for the treatment of advanced solid tumors, including mCRC, has completed patient enrollment and awaits final results.

The REGONIVO study has improved the pattern of immunotherapy in pMMR colorectal cancer and demonstrated the feasibility of modulating the tumor immune microenvironment to improve the effectiveness of immunotherapy [26]. Currently, there are several clinical studies targeting the modulation of TME to improve CRC immunotherapy effects. PB101 is a glycosylated decoy receptor with a high affinity for VEGF-A. It inhibits tumor growth by inhibiting angiogenesis and enhancing CD8^+^ T cell infiltration, in coordination with anti-PD-L1 antibodies [132]. A clinical trial (NCT06075849) investigating PB101 as a monotherapy for the treatment of advanced solid tumors, including mCRC, is currently ongoing. TTX-030 is a CD39 inhibitor that not only promotes the proliferation of effector T cells and activates NK cells but also inhibits the function of immunosuppressive cells, such as MDSCs and Treg cells, and induces macrophages to switch to a pro-inflammatory phenotype [133]. A clinical trial (NCT04306900) investigating TTX-030 in combination with immunotherapy and/or chemotherapy for the treatment of advanced solid tumors, including mCRC, has completed recruitment. There are also ongoing clinical studies targeting key immune inhibitory cell factors or receptors in the TME, such as CCR2/CCR5 (NCT03184870), CXCL12 (NCT03168139), CXCR1/2 (NCT04599140). However, the final results of these clinical trials have not been reported yet.

In conclusion, based on the current design of clinical studies, targeting TAMs to inhibit tumor infiltration, targeting MDSCs to promote their apoptosis, targeting Tregs to induce their exhaustion, and targeting key immune inhibitory cell factors or receptors in the TME may be feasible strategies to improve the immunotherapy effects of CRC. We look forward to the results of clinical trials to confirm the feasibility of these strategies. As scientists continue to research, we believe that more and more strategies targeting the TME to improve CRC immunotherapy effects will be applied in clinical practice.

### 1.8 Limitations and challenges

Reviewing existing research, targeting tumor microenvironment-associated immunosuppressive cells to improve the efficacy of immunotherapy for colorectal cancer is a promising approach. However, current research still has limitations. Firstly, most studies are still in the preclinical stage, primarily focusing on cellular or animal models, which may not accurately reflect the tumor immune microenvironment. It is necessary to explore the use of new experimental techniques, such as organoid technology, to better simulate the tumor microenvironment in basic research. Secondly, the majority of current studies focus on eliminating or altering the functional state of immunosuppressive cells, it is necessary to evaluate the effects of combining immune checkpoint inhibitors with targeted modulation of immunosuppressive cells to improve immunotherapy for colorectal cancer. Lastly, as targeted immunotherapy against the tumor microenvironment-associated immunosuppressive cells is a potential therapeutic approach, exploring suitable combination therapies with immunotherapies is an area that requires further investigation.

Regarding existing biomarkers for immunotherapy in colorectal cancer, systematic elucidations have been provided by Hou Wanting et al. [134]. In the future, as targeted immunotherapy against tumor environment-associated immunosuppressive cells emerges as a novel approach, the exploration of new appropriate biomarkers is also an area that requires further investigation. For example, developing an immune cell tumor infiltration scoring system that integrates the abundance of different immunosuppressive cells for personalized immunotherapy holds great promise.

## 2 Conclusion and prospects

Currently, MSS/pMMR colorectal cancer is generally considered a “cold tumor” in terms of immunotherapy. The emergence of the REGONIVO study suggests the feasibility of targeting the TME to improve the immunotherapy effects in MSS/pMMR colorectal cancer patients. Past research has shown the presence of various immune suppressive cells in the TME, such as M2-type TAMs, MDSCs, Tregs, CAFs, TANs, and immature DCs. These immune suppressive cells inhibit tumor infiltration of effector T cells or induce T cell dysfunction by secreting immune suppressive factors, thus inhibiting the effectiveness of tumor immunotherapy. The studies cited in this review suggest that targeting immune suppressive cells in the TME to improve the immunotherapy effects of CRC is a feasible strategy, and there are ongoing clinical trials in this regard. While researchers have recognized the feasibility of modulating the tumor immune microenvironment to improve immunotherapy effects, there is still relatively limited research on targeting the TME to improve CRC immunotherapy effects. Future studies should conduct more clinical research to further explore the feasibility of this treatment strategy and identify optimal combination approaches (such as combining TME targeting with ICIs, vaccines, or adoptive cell immunotherapy). Furthermore, future research should strengthen basic research on targeting the TME to explore more potential therapeutic targets. Additionally, comprehensive scoring of immune suppressive cell infiltration in the TME to predict immunotherapy responses may be a future research direction.

**TAM:** 1. Regulating TAM polarization from M2 to M1 phenotype; 2. Inhibt TAM differentiation into M2 phenotype; 3. Inducing TAM polarization towards M1 phenotype to reactivate anti-tumor functions; 4. Suppressing TAM tumor infiltration by reducing-associated chemoattractants.

**CAF:** 1. Reduce the production of immunosuppressive molecules by CAFs; 2. Inhibit CAF proliferation or induce “normalization” of CAF.

**MDSC:** 1. Downregulate immunosuppressive functions of MDSCs; 2. Induce apoptosis of MDSCs to reduce accumulation in the tumor microenvironment.

**Treg:** 1. Inhibit Treg proliferation; 2. Promote Treg apoptosis to eliminate Treg infiltration in tumor tissues.

**TAN:**1. Induce differentiation of TAN from N2 to N1 phenotype; 2. Inhibit TAN differentiation into N2 phenotype.

DC: Targeting specific molecules or receptors to promote DC maturation and differentiation.

Related clinical research on targeting tumor microenvironment in colorectal cancer.

## Funding

This study was supported by the Bureau of Science and Technology of Ganzhou Municipality, No. GZ2021ZSF341.

## Data availability

Data sharing is not applicable to this article as no new data were created or analyzed in this study.

## CRediT authorship contribution statement

**Daoyang Zou:** Writing – original draft, Investigation, Data curation. **Xi Xin:** Writing – review & editing. **Yunxian Xu:** Writing – review & editing. **Huangzhen Xu:** Writing – review & editing. **Linyan Huang:** Writing – review & editing. **Tianwen Xu:** Writing – review & editing, Supervision, Project administration, Conceptualization.

## Declaration of competing interest

The authors declare that they have no known competing financial interests or personal relationships that could have appeared to influence the work reported in this paper.
